# Prediction of Symptoms and Evaluation of Surgical Indications After Birth Based on Tracheal Morphology of Double Aortic Arch

**DOI:** 10.1007/s00246-025-04153-4

**Published:** 2026-01-12

**Authors:** Takeshi Ikegawa, Akio Kato, Motoyoshi Kawataki, Yoshinori Inagaki, Katsuaki Toyoshima, Hideaki Ueda

**Affiliations:** 1 Department of Cardiology, Kanagawa Children’s Medical Center, 2-138-4 Mutsukawa, Minami-ku, Yokohama, Kanagawa Japan; 2 Department of Neonatology, Kanagawa Children’s Medical Center, Yokohama, Kanagawa Japan

**Keywords:** Prenatal diagnosis, Tracheal stenosis, Respiratory insufficiency, Surgical planning, Fetal echocardiography, X-Ray computed tomography

## Abstract

To establish objective criteria for predicting postnatal respiratory symptoms and determine surgical indications in fetuses with double aortic arch (DAA). This single-center, retrospective cohort study included fetuses diagnosed with DAA using fetal echocardiography and managed perinatally at Kanagawa Children’s Medical Center from January 2013–December 2024. Clinical data, fetal echocardiography, postnatal contrast-enhanced computed tomography (CT) findings, surgical intervention, and outcomes were reviewed. Narrowest tracheal diameter (NTD) Z score in late gestation and narrowest-to-reference tracheal area (NTA/RTA) ratio on postnatal CT were measured. Primary and secondary outcomes included respiratory symptoms shortly after birth and elective surgery, respectively. Groups were compared using t-tests, and the optimal cutoff values were determined using receiver operating characteristic (ROC) curve analysis. Reproducibility was assessed using intraclass correlation coefficient. Twenty-two patients were included: 5 symptomatic and 17 asymptomatic (12, prophylactic surgery; 5, observation). Symptomatic patients had significantly lower NTD Z scores (median − 1.8 vs. −1.2, *P* = 0.012) and NTA/RTA ratios (median 0.39 vs. 0.67, *P* < 0.001). NTD Z score ≤ − 1.7 (area under the ROC curve [AUC] 0.87; sensitivity 80%, specificity 88%) and NTA/RTA ratio ≤ 0.5 (AUC 0.94; sensitivity 100%, specificity 82%) were optimal thresholds for predicting early symptoms. In the asymptomatic group, NTA/RTA ratio ≥ 0.56 identified candidates for observation without surgery. The ICC values for both parameters exceeded 0.85, indicating excellent reproducibility. The prenatal NTD Z score and postnatal NTA/RTA ratio accurately predict postnatal respiratory symptoms in fetuses with DAA. These quantitative parameters may guide individualized perinatal management and surgical decision-making.

## Introduction

Double aortic arch (DAA) is a congenital cardiovascular anomaly in which both the left and right aortic arches persist, forming a vascular ring that encircles and compresses the trachea and esophagus [1]. The clinical presentation can vary notably; some neonates may present with severe respiratory symptoms immediately after birth, whereas others develop respiratory distress or dysphagia during infancy [2]. Surgical repair is the only definitive treatment for DAA. Prolonged vascular ring compression causes hypoplasia of the tracheal cartilage, potentially leading to tracheomalacia [1]. Therefore, prompt surgical intervention is recommended when symptoms are present, and all cases are managed surgically at the time of diagnosis because most cases are identified after the onset of respiratory symptoms [2]. However, diagnosis after symptom onset often delays treatment intervention [3], the ability to predict postnatal respiratory symptoms during the perinatal period could enable timely surgical planning and improved outcomes [4]. Recent advancements in fetal echocardiography and prenatal imaging have resulted in an increasing number of DAA cases being diagnosed in utero and have revealed that many prenatally diagnosed cases remain asymptomatic after birth [5, 6]. This finding suggests that not all cases require immediate surgical repair and highlights the need to re-evaluate management strategies for fetuses with DAA. This study aimed to develop criteria for predicting postnatal symptom onset and determining surgical indications in fetuses with DAA, thereby facilitating perinatal management and optimizing surgical planning.

## Methods

### Study Population

This single-center, retrospective cohort study included fetuses diagnosed with DAA using fetal echocardiography during the perinatal period at Kanagawa Children’s Medical Center, Kanagawa, Japan. Fetuses diagnosed with DAA between January 2013 and December 2024 who underwent perinatal management at our institution were included. Data on fetal echocardiography, diagnosis, associated complications, postnatal surgical intervention, outcomes, and postnatal contrast-enhanced computed tomography (CT) were retrospectively extracted from medical records. Given that fetal echocardiographic parameters are expected to change during the course of pregnancy, data obtained in late gestation (gestational age ≥ 28 weeks) were collected to reflect postnatal outcomes more accurately. Patients whose postnatal evaluation did not confirm a final diagnosis of DAA, as well as those with comorbidities potentially contributing to airway stenosis unrelated to the vascular ring, were excluded. This study was approved by the Research Ethics Committee of the Kanagawa Children’s Medical Center (approval number: 175-7). Due to the retrospective and observational nature of the investigation, the risk associated with participation in this study was considered low, and informed consent could not be obtained from each participant. Therefore, the requirement for informed consent was waived, and an opt-out approach was applied (patients were considered to have agreed to participate in this study unless they declined). Information regarding this study is available on the Kanagawa Children’s Medical Center website (https://kcmc.kanagawa-pho.jp/data/media/01_soumu/rinri/rin-circulation/circulation2505.pdf).

### Data Collection and Measurement Method

Prenatal echocardiograms were obtained using a GE Voluson E8 (with a 2–5-MHz C1-5D convex probe or a 2–6-MHz RM6C convex probe), GE Voluson E10 (with a 3–9-MHz C2-9D convex probe or a 1–7-MHz RM6C convex probe), or GE Voluson Expert 22 (with a 3–9-MHz C2-9D convex probe or a 1–7-MHz RM6C convex probe) ultrasound system (GE Healthcare, Zipf, Austria). Postnatal CT examinations were performed using either an Aquilion 64 or Aquilion ONE CT scanner (Canon Medical Systems, Otawara, Japan). All CT scans were contrast-enhanced, and the contrast medium dose was calculated based on body weight (1.5 mL/kg) and administered with a power injector for 20–25 s. All tracheal measurements were performed three times by a single observer (T.I.), who was blinded to the patients’ clinical and follow-up data. The mean of the three measurements was used for analysis. For tracheal morphometry, the “narrowest tracheal site” was defined as the segment encircled by the vascular ring, whereas the “reference tracheal site” was defined as the segment at the level of the clavicles, which typically shows a relatively constant diameter and no stenosis.

Cine-loop images acquired during late gestation were analyzed using the measurement function of the GE Voluson Expert 22. The narrowest tracheal diameter (NTD) was measured on the sagittal plane, and the “NTD Z score” was calculated according to a previously published method [7]. Specifically, the Z-score was calculated as.

$$ NTD{\text{ }}Z{\text{ }}score\, = \,\frac{{{\mathrm{Measured~NTD~}}\left[ {{\mathrm{mm}}} \right]~ - {\mathrm{~Predicted~NTD~}}\left[ {{\mathrm{mm}}} \right]}}{{{\mathrm{~Predicted~SD}}}} $$

$$ \begin{gathered} Predicted{\text{ }}NTD{\text{ }}\left[ {mm} \right] \hfill \\ \quad = \,\left( { - {\mathrm{1}}.{\mathrm{423}}\, \times \,{\text{Gestational age }}\left[ {{\mathrm{weeks}}} \right]} \right)\, + \,0.{\mathrm{147}} \hfill \\ \end{gathered} $$

$$ \begin{gathered} Predicted{\text{ }}SD \hfill \\ \quad {\mkern 1mu} = {\mkern 1mu} \sqrt {\frac{\pi }{2}} {\mkern 1mu} \times {\mkern 1mu} 0.0{\mathrm{29}}{\mkern 1mu} \times {\mkern 1mu} {\text{Gestational age }}\left[ {{\mathrm{weeks}}} \right] \hfill \\ \end{gathered} $$

The CT images were analyzed using the RAPID-EyeCore image analysis platform (Canon Medical Systems, Otawara, Japan). The regions of interest were manually traced, and the narrowest tracheal area (NTA) and reference tracheal area (RTA) were measured on axial images. The CT display parameters were standardized to a soft-tissue window (window level: 40 HU; window width: 400 HU) [8]. The NTA/RTA ratios were then calculated.

**Fig. 1:**
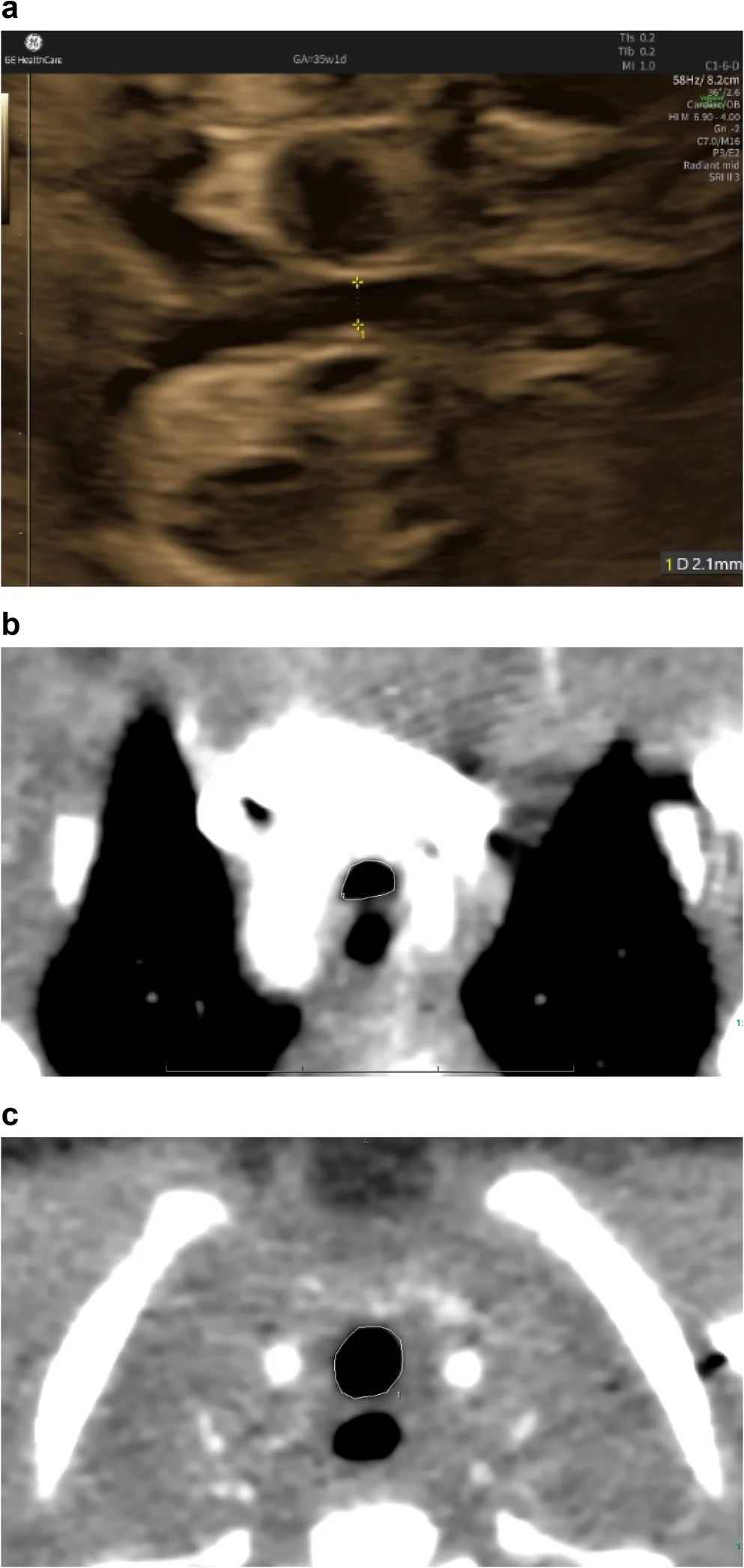
**a** Sagittal view of a late-gestation fetal echocardiographic image. The narrowest tracheal diameter surrounded by the vascular ring was measured using the measurement function of the GE Voluson Expert 22 system, (GE Healthcare, Zipf, Austria)., **b** Axial postnatal computed tomography, (CT) image. The cross-sectional area of the narrowest tracheal segment encircled by the vascular ring was measured by manually tracing the region of interest, (ROI) using RAPID-EyeCore software, (Canon Medical Systems Corporation, Otawara, Japan)., **c** Axial postnatal CT image. The cross-sectional area of a reference tracheal segment located at the level of the clavicles, where no narrowing was present and the morphology was relatively uniform, was measured using the same method as in, (**b**)

The NTD Z-score obtained from prenatal echocardiography and the NTA/RTA ratio obtained from postnatal CT were used for subsequent analyses. Representative examples of fetal echocardiography and CT are shown in Fig. 1.

### Statistical Analysis

The primary outcome was the appearance of clinical signs shortly after birth, including stridor, wheezing, coughing, or dyspnea [5]. The secondary outcome was elective surgery after birth. According to the primary outcome, patients were classified into two groups: symptomatic and asymptomatic. Based on the secondary outcomes, the patients were further divided into surgery and observation groups. Patients with postnatal symptoms who underwent emergency surgery were classified into the symptomatic group, whereas those without postnatal symptoms were classified into the asymptomatic group. The asymptomatic group was further subdivided into an asymptomatic observation group (no surgery) and an asymptomatic surgery group (prophylactic semi-urgent surgery). For univariate analyses, Student’s *t*-test was used when normality and homogeneity of variance were confirmed. Receiver operating characteristic (ROC) curve analysis was performed to determine the cutoff values for continuous variables. The optimal cutoff value was calculated based on the Youden index, which maximizes the sum of sensitivity and specificity. Correlation analyses were conducted using Pearson product-moment correlation coefficient when both variables followed a normal distribution. To evaluate the intraobserver agreement, a second measurement by the same observer was performed after a certain interval. To evaluate the interobserver agreement, another observer (A.K.) repeated the measurements. Intra- and interobserver reproducibility were assessed using intraclass correlation coefficients (ICC). Statistical analyses were performed using JMP v18 (SAS Institute Inc., Cary, NC, USA), and R v4.5.1 (R Foundation for Statistical Computing, Vienna, Austria); a *P* value < 0.05 was considered statistically significant.

## Results

### Baseline Characteristics

Overall, 22 patients were included in the analysis. Of these, 5 and 17 were classified into the symptomatic and asymptomatic groups, respectively (5 in the asymptomatic observation group and 12 in the asymptomatic surgery group). Table 1 summarizes the clinical characteristics and outcomes of the 22 cases. The types of DAA were classified into complete DAA (cDAA), in which both arches were patent, and incomplete DAA (iDAA), in which one arch was partially closed. There were 12 cDAA and 10 cases iDAA cases. All iDAA cases were right aortic arch (RAA) dominant with regression of the left aortic arch (LAA). Associated anomalies were detected in three patients: one had a common arterial trunk with persistent left superior vena cava and ASXL2 mutation, one had a ventricular septal defect with 22q11.2 deletion syndrome, and one had isolated persistent left superior vena cava. Postnatal contrast-enhanced CT was performed in all 22 patients. In the symptomatic group (*n* = 5), the median age at symptom onset was 3 days (range, 0–5 days). In the asymptomatic surgery group (*n* = 12), the median age at surgery was 26.5 days (range, 8–114 days) and no symptoms were observed prior to surgery. During the follow-up period (excluding the preoperative period in the symptomatic group), all 22 patients remained asymptomatic, with a median follow-up of 34 months (range, 12–151 months). Only one case developed postoperative recurrent laryngeal nerve palsy as a complication.

**Table 1:** Clinical characteristics in fetuses diagnosed prenatally with double aortic arch

Case	Group	Sub Group	DAA type	Chromosome abnormality	Heart Complication	GA at fetal echo performed (weeks)	Age at CT performed (days)	Age at symptom onset (days)	Operative age (days)	Postoperative complication	Follow-up period (months)
1	Symptomatic	N/A	cDAA	No	No	35	3	3	5	No	32
2	Symptomatic	N/A	iDAA	No	No	35	5	4	8	No	31
3	Symptomatic	N/A	iDAA	No	No	36	5	0	7	No	26
4	Symptomatic	N/A	iDAA	No	No	35	3	5	12	No	21
5	Symptomatic	N/A	cDAA	ASXL2 mutation	Truncus, PLSVC	35	0	0	0	No	44
6	Asymptomatic	Observation	iDAA	No	No	38	4	N/A	N/A	N/A	128
7	Asymptomatic	Observation	iDAA	No	No	34	1	N/A	N/A	N/A	69
8	Asymptomatic	Observation	cDAA	No	No	35	2	N/A	N/A	N/A	36
9	Asymptomatic	Observation	cDAA	No	No	36	6	N/A	N/A	N/A	23
10	Asymptomatic	Observation	iDAA	No	No	32	30	N/A	N/A	N/A	21
11	Asymptomatic	Surgery	cDAA	22q11.2 DS	VSD	35	23	N/A	24	No	128
12	Asymptomatic	Surgery	cDAA	No	PLSVC	35	4	N/A	32	No	73
13	Asymptomatic	Surgery	iDAA	No	No	39	4	N/A	14	No	14
14	Asymptomatic	Surgery	iDAA	No	No	35	5	N/A	35	No	82
15	Asymptomatic	Surgery	cDAA	No	No	37	2	N/A	22	No	69
16	Asymptomatic	Surgery	cDAA	No	No	29	49	N/A	87	No	27
17	Asymptomatic	Surgery	cDAA	No	No	31	3	N/A	27	No	57
18	Asymptomatic	Surgery	iDAA	No	No	38	9	N/A	26	No	38
19	Asymptomatic	Surgery	cDAA	No	No	33	2	N/A	8	No	18
20	Asymptomatic	Surgery	iDAA	No	No	35	4	N/A	27	RLNP	150
21	Asymptomatic	Surgery	cDAA	No	No	34	6	N/A	25	No	12
22	Asymptomatic	Surgery	cDAA	No	No	33	8	N/A	114	No	22

*DAA* Double aortic arch, *GA* Gestational age, *CT* Not applicable, *cDAA* Complete double aortic arch, *iDAA* Incomplete double aortic arch, *PLSVC* Persistent left superior vena cava, *22q11.2 DS* 22q11.2 Deletion syndrome, *VSD* Ventricular septal defect, *RLNP* Recurrent laryngeal nerve palsy

### Group Comparison and Correlation Between NTD Z Score and NTA/RTA Ration

**Fig. 2:**
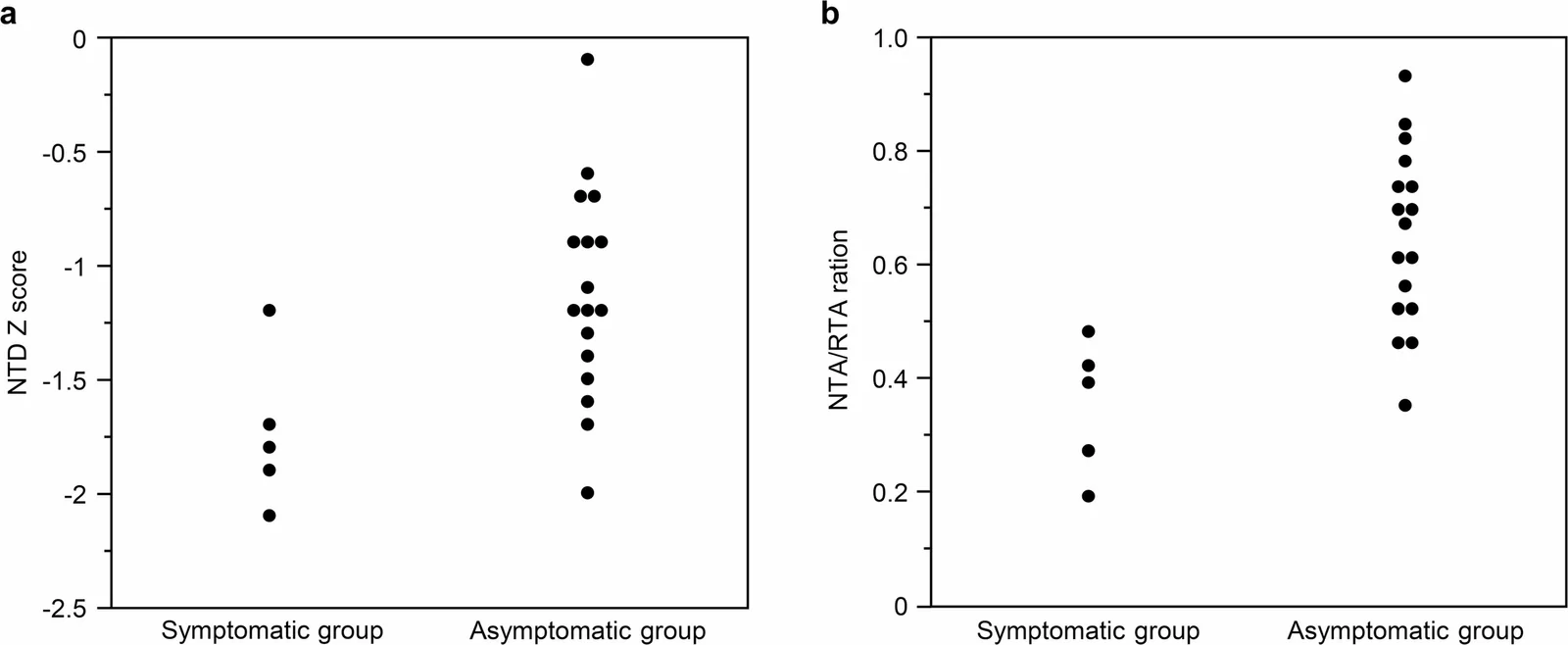
**a** Distribution of NTD Z scores in the symptomatic and asymptomatic groups. The median NTD Z score was − 1.8, (range, − 2.1 to − 1.2) in the symptomatic group and − 1.2, (range, − 2.0 to − 0.1) in the asymptomatic group., **b** Distribution of NTA/RTA ratios in the symptomatic and asymptomatic groups. The median NTA/RTA ratio was 0.39, (range, 0.19–0.48) in the symptomatic group and 0.67, (range, 0.35–0.93) in the asymptomatic group. NTD, narrowest tracheal diameter; NTA/RTA, narrowest-to-reference tracheal area ratio

Table 2 summarizes the tracheal morphometric parameters of the 22 cases, and Fig. 2 illustrates the distribution of the NTD Z-scores and NTA/RTA ratios. The median fetal echocardiographic NTD Z-score was − 1.8 (range, − 2.1 to − 1.2) in the symptomatic group and − 1.2 (range, − 2.0 to − 0.1) in the asymptomatic group. Two-sided student’s t-test revealed a statistically significant difference between the symptomatic and asymptomatic groups (*P* = 0.012). The median NTA/RTA ratio was 0.39 (range, 0.19–0.48) in the symptomatic group and 0.67 (range, 0.35–0.93) in the asymptomatic group. Two-sided Student’s t-test also demonstrated a statistically significant difference between the two groups (*P* = 0.0009).

**Table 2:** Tracheal morphometric parameters in fetuses diagnosed prenatally with double aortic arch

Case	Group	Subgroup	NTD on fetal echo (mm)	NTD Z score on fetal echo	NTA on CT (mm^2^)	RTA on CT (mm^2^)	NTA/RTA ration on CT
1	Symptomatic	N/A	1.0	-1.9	4.5	23.6	0.19
2	Symptomatic	N/A	1.1	-1.8	7.3	26.7	0.27
3	Symptomatic	N/A	1.4	-1.7	9.9	25.8	0.39
4	Symptomatic	N/A	2.1	-1.2	9.7	22.9	0.42
5	Symptomatic	N/A	0.7	-2.1	11.8	24.4	0.48
6	Asymptomatic	Observation	2.2	-1.2	18.6	25.1	0.74
7	Asymptomatic	Observation	1.9	-1.2	12.3	22.1	0.56
8	Asymptomatic	Observation	2.4	-0.9	17.9	26.6	0.67
9	Asymptomatic	Observation	2.0	-1.3	13.5	19.8	0.68
10	Asymptomatic	Observation	2.5	-0.6	23.3	28.3	0.82
11	Asymptomatic	Surgery	2.4	-0.9	28.2	30.2	0.93
12	Asymptomatic	Surgery	2.4	-0.9	22.8	29.2	0.78
13	Asymptomatic	Surgery	2.6	-1.1	19.1	32.0	0.60
14	Asymptomatic	Surgery	1.5	-1.5	11.1	23.6	0.47
15	Asymptomatic	Surgery	2.0	-1.4	14.8	21.8	0.68
16	Asymptomatic	Surgery	1.4	-1.2	11.3	25.1	0.45
17	Asymptomatic	Surgery	2.2	-0.7	17.5	23.9	0.73
18	Asymptomatic	Surgery	1.0	-2.0	13.4	21.5	0.62
19	Asymptomatic	Surgery	1.2	-1.6	8.1	23.4	0.35
20	Asymptomatic	Surgery	1.3	-1.7	11.7	22.4	0.52
21	Asymptomatic	Surgery	3.5	-0.1	16.1	31.0	0.52
22	Asymptomatic	Surgery	2.4	-0.7	27.3	33.0	0.83

*NTD* Narrowest tracheal diameter, *NTA* Narrowest tracheal area, *CT* Computed tomography, *RTA* Reference tracheal area, *N/A* Not applicable

### ROC Curve Analysis

**Fig. 3:**
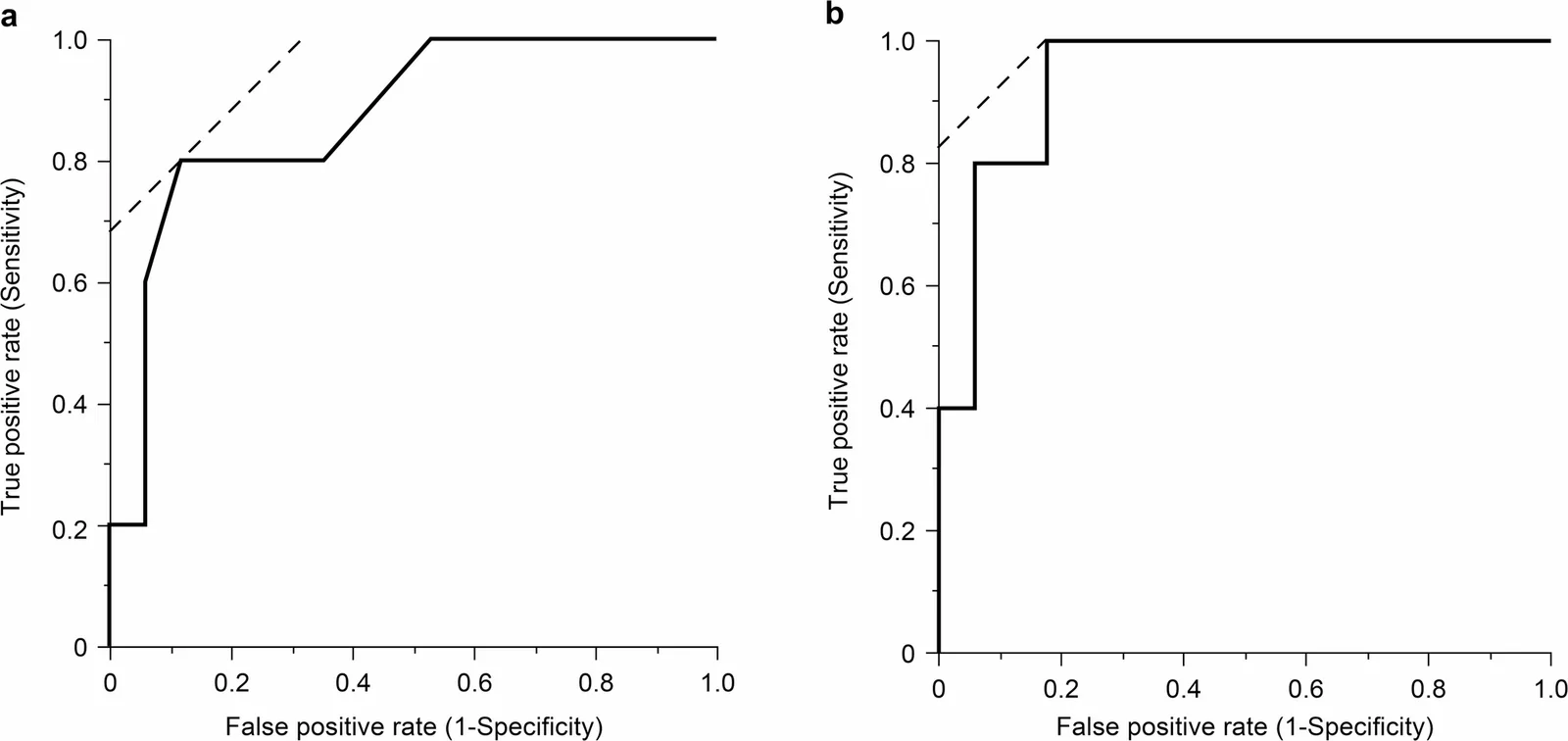
Receiver-operating-characteristics curve analyses of (**a**) the NTD Z score and (**b**) the NTA/RTA ratio for predicting postnatal symptoms in the symptomatic and asymptomatic groups. An NTD Z score ≤−1.7 and an NTA/RTA ratio ≤0.5 were identified as thresholds for predicting the occurrence of respiratory symptoms shortly after birth. NTD. narrowest tracheal diameter; NTA/RTA, narrowest-to-reference tracheal area ratio

Figure 3 summarizes the ROC curve analysis of the fetal echocardiographic NTD Z-score and postnatal CT-derived NTA/RTA ratio. An NTD Z score ≤ − 1.7 and an NTA/RTA ratio ≤ 0.5 were identified as criteria for predicting the occurrence of clinical symptoms shortly after birth. The cutoff value of the NTD Z score showed high sensitivity (80.0%) and specificity (88.2%), with an area under the ROC curve (AUC) of 0.87 (95% confidence interval [CI]: 0.69–1.00), serving as a threshold to prenatally identify high-risk cases. In contrast, the cutoff value of the NTA/RTA ratio demonstrated excellent sensitivity (100%) and high specificity (82.4%), with an AUC of 0.94 (95% CI: 0.84–1.00), representing a threshold at which urgent postnatal surgery should be considered. In addition, a correlation analysis was performed between the NTD Z-score and NTA/RTA ratio in all 22 cases. Using Pearson product–moment correlation coefficient, a moderately positive correlation was observed between the two variables (*r* = 0.59 [95% CI: 0.23–0.81], *P* = 0.0037).

### Subgroup Analysis of the Asymptomatic Group for Surgical Decision-Making

**Fig. 4:**
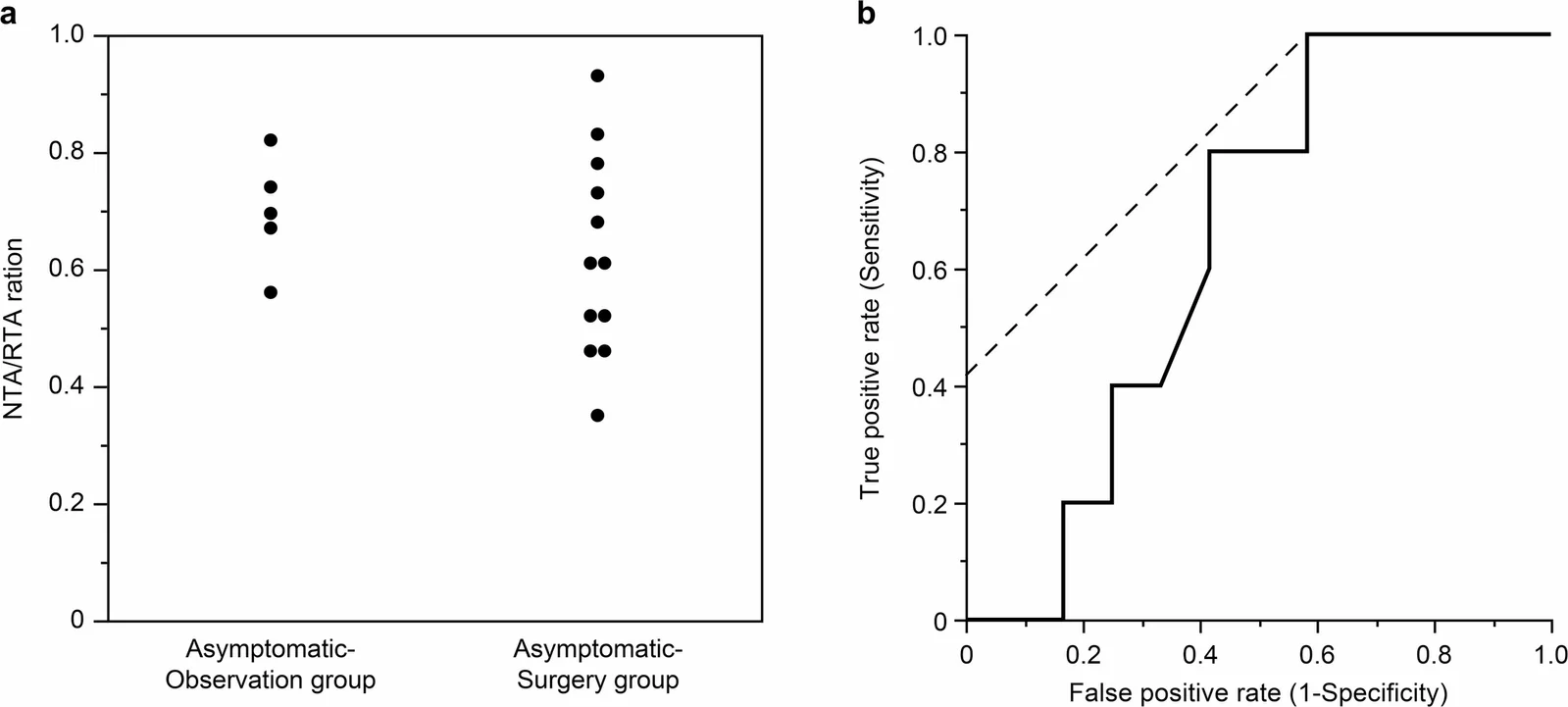
Subgroup analysis of the asymptomatic group comparing the asymptomatic-observation group (no surgery) and the asymptomatic-surgery group (prophylactic semi-urgent surgery). **a** Distribution of NTA/RTA ratios and (**b**) Receiver operating characteristic (ROC) curve analysis of the NTA/RTA ratio. An NTA/RTA ratio ≥ 0.56 was identified as the threshold for considering observation without elective surgery. Although this cut-off showed high sensitivity (100%), its specificity was low (41.7%), with an area under the ROC curve (AUC) of 0.64. NTD. narrowest tracheal diameter; NTA/RTA, narrowest-to-reference tracheal area ratio; AUC, area under the receiver operating characteristic curve

Five patients in the asymptomatic observation group were conservatively managed without surgery. There were no explicit criteria for this decision. Surgery was withheld when there was no significant tracheal narrowing on postnatal CT and/or when this approach was preferred by the family. Therefore, we examined whether elective surgery should be considered for patients who remain asymptomatic shortly after birth. Figure 4 summarizes the distribution of NTA/RTA ratios and ROC curve analysis of the 17 patients in the asymptomatic group. The median NTA/RTA ratio was 0.68 (range, 0.56–0.82) in the asymptomatic-observation group and 0.61 (range, 0.35–0.93) in the asymptomatic-surgery group. An NTA/RTA ratio ≥ 0.56 was identified as the threshold for considering observation without elective surgery. The cutoff value demonstrated high sensitivity (100%) but low specificity (41.7%), with an area under the ROC curve (AUC) of 0.64 (95% CI: 0.37–0.91).

### Intraclass Correlation Coefficients

The ICC for measuring the fetal echocardiographic NTD Z score and postnatal CT-derived NTA/RTA ratio were evaluated using 10 randomly selected cases. The intraobserver agreement for the NTD Z-score was 0.95 (95% CI: 0.81–0.99), and the interobserver agreement was 0.87 (95% CI: 0.57–0.96). In contrast, the intraobserver agreement for the NTA/RTA ratio was 0.99 (95% CI: 0.98–1.00), and the interobserver agreement was 0.95 (95% CI: 0.80–0.99). Both the measurements demonstrated excellent reproducibility.

## Discussion

This study demonstrated a significant correlation between the fetal tracheal NTD Z score and the postnatal CT-derived NTA/RTA ratio, showing that both parameters can predict the occurrence of postnatal respiratory symptoms in fetuses with DAA with high accuracy. In particular, an NTA/RTA ratio ≤ 0.5 provided high sensitivity and specificity, suggesting that it may serve as an objective criterion for determining the need for urgent surgical intervention.

A DAA is a vascular ring that most likely causes respiratory symptoms shortly after birth, and surgical intervention is traditionally performed after symptom onset [2]. With an increasing number of prenatal diagnoses, cases with an asymptomatic postnatal course have also been reported [5, 6], highlighting the importance of evaluating tracheal morphology and predicting symptom onset during the perinatal period. The tracheal internal diameter Z score relative to gestational age measured by fetal ultrasound [7] and a smaller double-arch pinch angle on prenatal ultrasound are associated with postnatal symptoms [4], and postnatal CT can qualitatively delineate the site and severity of tracheal obstruction [9, 10], However, no previous studies have quantitatively evaluated the tracheal morphometric parameters using postnatal CT and directly correlated them with postnatal clinical symptoms. To our knowledge, this is the first study to quantitatively evaluate tracheal morphology from the prenatal to postnatal period and to propose predictive thresholds directly associated with postnatal outcomes.

Because the trachea and esophagus are completely encircled by double aortic arches in DAA, circumferential compression can cause tracheal narrowing and other symptoms [1]. The NTD Z score in late gestation and the NTA/RTA ratio on postnatal CT can be used to quantitatively evaluate these morphological changes, reflecting the risk of symptom manifestation when ventilatory demand increases after birth. Among these parameters, the NTA/RTA ratio on postnatal CT showed a strong association with the occurrence of postnatal respiratory symptoms. Although the trachea grows throughout gestation [11], previous studies have shown that tracheal growth accelerates after birth, particularly between 1 month and 4 years of age [12]. Our findings demonstrate that tracheal morphometry during late gestation sufficiently correlates with postnatal tracheal morphology and can be used as a reliable prenatal predictor of postnatal symptoms.

Based on of the thresholds identified in this study, we propose a practical perinatal management strategy. If the NTD Z score in late gestation is ≤ − 1.7, there is a risk of postnatal respiratory symptoms, and perinatal planning should include preparations for potential airway management at delivery. Postnatally, contrast-enhanced CT should be performed to evaluate tracheal narrowing, and if the NTA/RTA ratio is ≤ 0.5, early surgical intervention should be considered given the high risk of symptom onset.

There is still no clear consensus regarding surgical intervention for asymptomatic patients with DAA. One report recommended elective repair at 6–9 months of age, even in patients with minimal or no symptoms [13]. However, clinical observation without surgery is a legitimate option for asymptomatic patients with DAA with favorable long-term outcomes [14]. Based on the results of the present study, patients with a relatively large NTA/RTA ratio may remain asymptomatic over the long term without surgery, which supports the findings of the aforementioned report [14]. In this study, an NTA/RTA ratio ≥ 0.56 may serve as a threshold for considering observation and avoiding elective surgery. Although surgical repair for DAA is generally a low-risk procedure, postoperative complications may occur. In our series, one case of recurrent laryngeal nerve palsy was observed in 17 surgical patients. Previous reports have described postoperative chylothorax in 9% of patients and the need for reoperation in 1% [15]. Thus, the ability to safely avoid unnecessary surgeries may have important clinical implications.

Because the morphometric parameters were measured manually, concerns regarding reproducibility may have arisen. ICC values > 0.75 are generally considered to indicate good reliability; values > 0.90 indicate excellent reliability [16]. In our study, the intra- and inter-observer ICCs for both the NTD Z-score and NTA/RTA ratio exceeded 0.85, confirming high reproducibility. Therefore, these parameters can be reliably applied to other institutions.

This study had several limitations. First, this was a retrospective study and measurements were obtained using multiple ultrasound and CT scanners rather than a single standardized system; therefore, measurement errors could not be fully assessed. Second, the primary outcome was limited to early postnatal respiratory symptoms; later-onset symptoms and swallowing-related symptoms were not assessed. In the asymptomatic surgery group, 12 patients underwent elective surgery at a median age of 26.5 days (range, 8–114 days), and they did not exhibit symptoms such as stridor, wheezing, cough, dyspnea, or dysphagia. Some of these patients may have developed symptoms later if surgery had not been performed. In the asymptomatic observation group, 5 patients were followed up until a median age of 36 months (range, 21–128 months), and they remained asymptomatic without stridor, wheezing, cough, dyspnea, or dysphagia. Previous studies have reported a median age of symptom onset of 5 months (range, 0 days to 10.3 years) [15]. Even in cases in which symptoms are absent shortly after birth, careful long-term follow-up is required, and surgery should be considered if symptoms develop. Third, the five patients in the asymptomatic observation group were not selected for observation based on a predefined protocol but rather on clinical judgment. Although all five had an NTA/RTA ratio ≥ 0.56 and remained asymptomatic during long-term follow-up, the sample size was small and the cut-off value of 0.56 should be interpreted with caution. Finally, this study was conducted on a small cohort, which limited its statistical power. Larger multicenter studies are warranted to validate the proposed thresholds.

In conclusion, both prenatal and postnatal tracheal morphometric parameters can accurately predict postnatal respiratory symptoms in fetuses with DAA. These objective data may offer valuable insights for managing perinatal care and surgical planning in this patient population.

## Data Availability

The datasets generated and/or analyzed during the current study are not publicly available due to patient privacy concerns and institutional data use regulations but are available from the corresponding author on reasonable request and with permission from the institutional review board.
