# Correction to: Prediction of Symptoms and Evaluation of Surgical Indications After Birth Based on Tracheal Morphology of Double Aortic Arch

**DOI:** 10.1007/s00246-026-04209-z

**Published:** 2026-03-25

**Authors:** Takeshi Ikegawa, Akio Kato, Motoyoshi Kawataki, Yoshinori Inagaki, Katsuaki Toyoshima, Hideaki Ueda

**Affiliations:** 1 Department of Cardiology, Kanagawa Children’s Medical Center, 2-138-4 Mutsukawa, Minami-ku, Yokohama, Kanagawa Japan; 2 Department of Neonatology, Kanagawa Children’s Medical Center, Yokohama, Kanagawa Japan

**Correction to: Pediatric Cardiology**

**https://doi.org/10.1007/s00246-025-04153-4**.

The authors wish to correct the following errors in the above-mentioned article. The authors apologize for these errors and provide the corrected information below:

1. Correction of the regression formula description

An error was identified in the written description of the regression equation for the predicted normal tracheal diameter (NTD). Importantly, this transcription error was limited to the written description in the manuscript, and did not affect the statistical analyses as all analyses were conducted using the correct regression formula. As such, the calculated results and study conclusions remain unchanged. Further, this issue was independent of the additional computational revisions described below.

2. Additional calculation revisions identified during reanalysis.

During the verification of the calculations, two additional issues requiring correction were identified. When calculating the tracheal Z score, gestational age was entered as completed weeks only, rather than including fractional weeks (e.g., 35+3/7 weeks instead of 35 weeks). In calculating the predicted standard deviation √2 was used instead of √(π/2) defined in the formula. Following correction, all NTD Z-scores and related statistical analyses were recalculated.

These revisions affected the analyses involving NTD Z-scores, including group comparisons, ROC analysis, correlation analysis, and ICC assessment. However, these corrections did not influence the statistical significance of the results or the overall conclusions of the study.

A comprehensive summary of all corrections is provided in the Correction Table below, including updates to:

**Table:** 

Section of the manuscript	Incorrect	Correct
Abstract	NTD Z scores (median − 1.8 vs. −1.2, *P* = 0.012)	NTD Z scores (median − 2.1 vs. −1.4, *P* = 0.014)
Abstract	NTD Z score ≤ − 1.7 (area under the ROC curve [AUC] 0.87…	NTD Z score ≤ − 1.9 (area under the ROC curve [AUC] 0.85…
Methods, Data Collection and Measurement Method section	Predicted NTD [mm] = (− 1.423 × Gestational age [weeks]) + 0.147	Predicted NTD [mm] = − 1.423 + 0.147 × Gestational age [weeks]
Results, Group Comparison and Correlation Between NTD Z Score and NTA/RTA Ratio section	NTD Z-score was − 1.8 (range, − 2.1 to − 1.2) in the symptomatic group and − 1.2 (range, − 2.0 to − 0.1)	NTD Z-score was − 2.1 (range, − 2.4 to − 1.3) in the symptomatic group and − 1.4 (range, − 2.3 to − 0.1)
Results, Group Comparison and Correlation Between NTD Z Score and NTA/RTA Ratio section	asymptomatic groups (*P* = 0.012)	asymptomatic groups (*P* = 0.014)
Results, ROC Curve Analysis section	NTD Z score ≤ − 1.7	NTD Z score ≤ − 1.9
Results, ROC Curve Analysis section	with an area under the ROC curve (AUC) of 0.87 (95% confidence interval [CI]: 0.69–1.00)	with an area under the ROC curve (AUC) of 0.85 (95% confidence interval [CI]: 0.63–1.00)
Results, ROC Curve Analysis section	the two variables (*r* = 0.59 [95% CI: 0.23–0.81], *P* = 0.0037)	the two variables (*r* = 0.58 [95% CI: 0.21–0.81], *P* = 0.0046)
Results, Intraclass Correlation Coefficients section	the NTD Z-score was 0.95 (95% CI: 0.81–0.99)	the NTD Z-score was 0.93 (95% CI: 0.75–0.98)
Results, Intraclass Correlation Coefficients section	the interobserver agreement was 0.87 (95% CI: 0.57–0.96)	the interobserver agreement was 0.87 (95% CI: 0.58–0.97)
Discussion section (paragraph beginning with “Based on of the thresholds…”)	the NTD Z score in late gestation is ≤ − 1.7	the NTD Z score in late gestation is ≤ − 1.9
Figure 2a	Previous distribution of NTD Z-scores	Updated distribution based on recalculated Z-scores
Figure 2 legend	NTD Z score was − 1.8 (range, − 2.1 to − 1.2) in the symptomatic group and − 1.2 (range, − 2.0 to − 0.1)	NTD Z score was − 2.1 (range, − 2.4 to − 1.3) in the symptomatic group and − 1.4 (range, − 2.3 to − 0.1)
Figure 3a	Previous ROC curve for NTD Z-score	Revised ROC curve based on recalculated NTD Z-scores
Figure 3 legend	NTD Z score ≤ − 1.7	NTD Z score ≤ − 1.9
Table 1, “GA at fetal echo performed (weeks)”	Gestational age reported in completed weeks only	Gestational age reported in weeks and days
Table 1, Footnote	*CT* Not applicable	*CT*, computed tomography; *N/A*, not applicable
Table 2, “NTD Z score on fetal echo”	Previously reported Z score values	Recalculated and updated Z score values

Incorrect Fig. 2.

**Figure:**
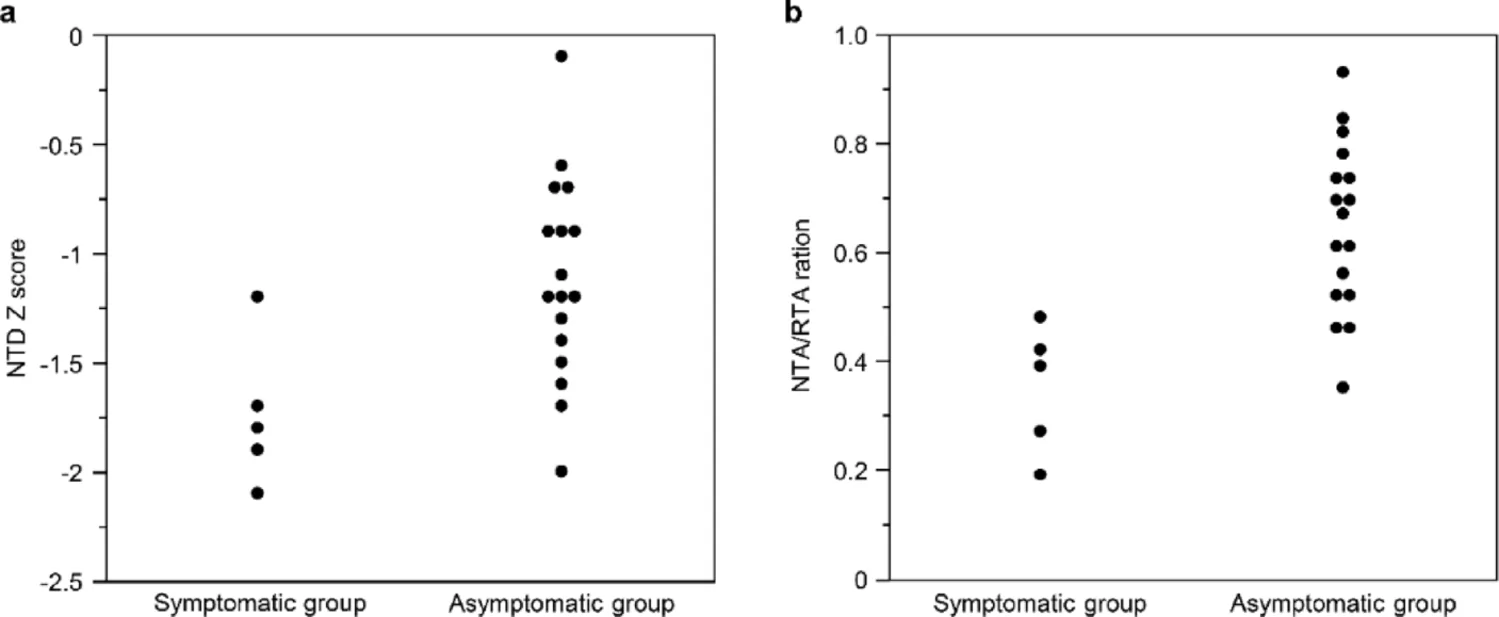

Correct Fig. 2.

**Figure:**
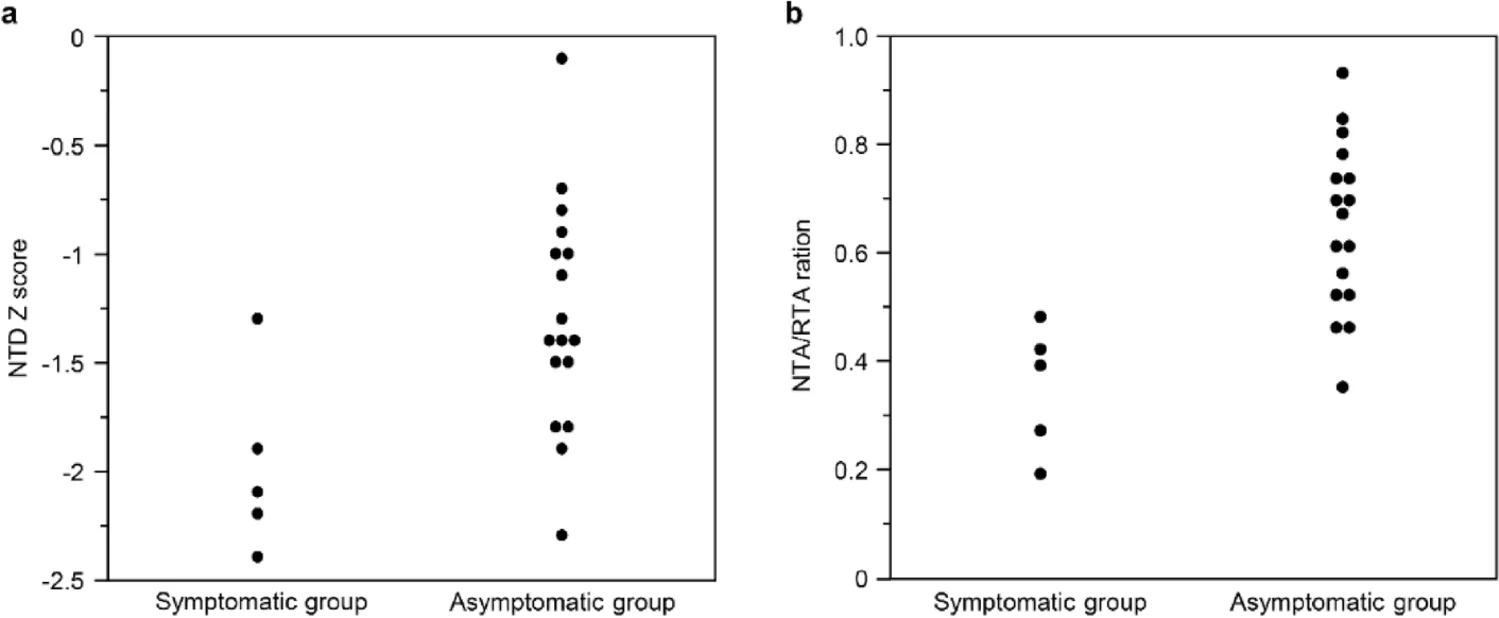

Incorrect Fig. 3.

**Figure:**
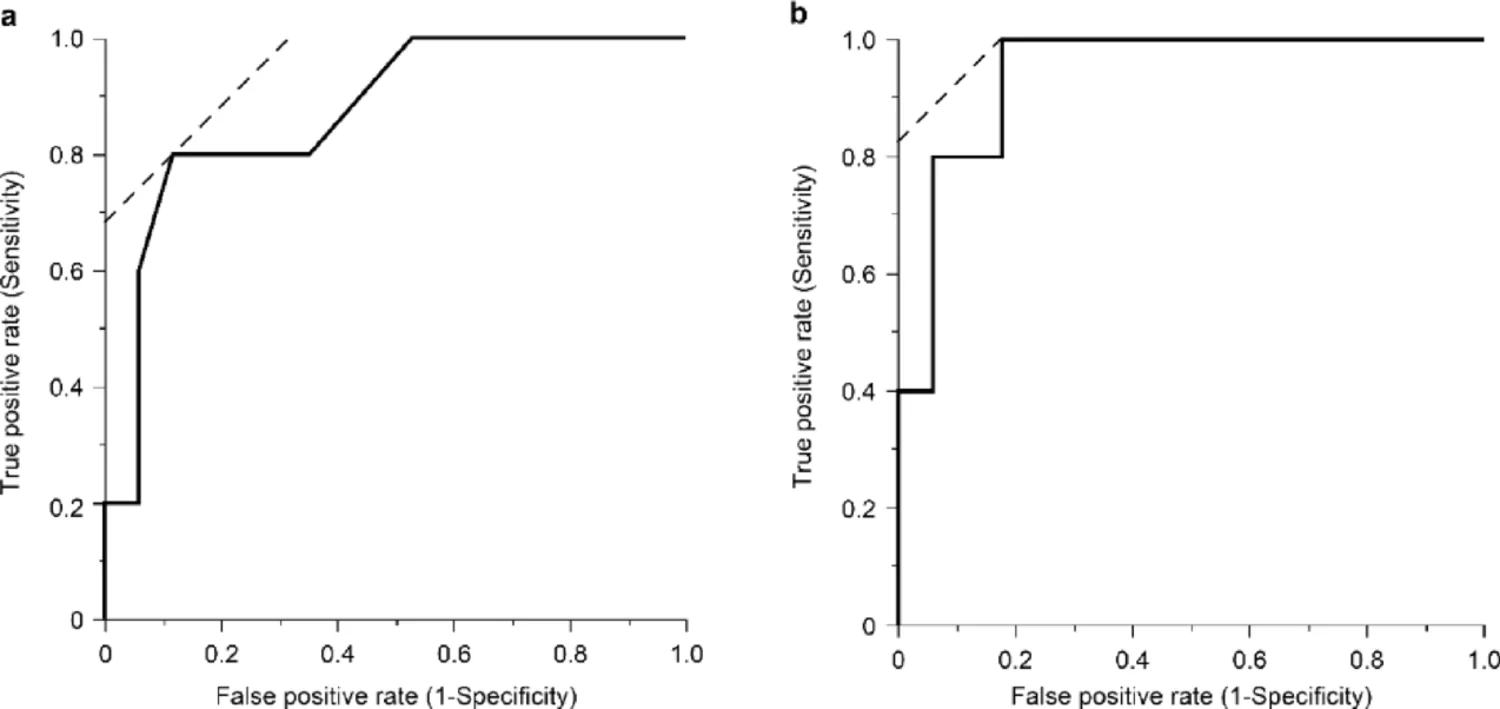

Correct Fig. 3.

**Figure:**
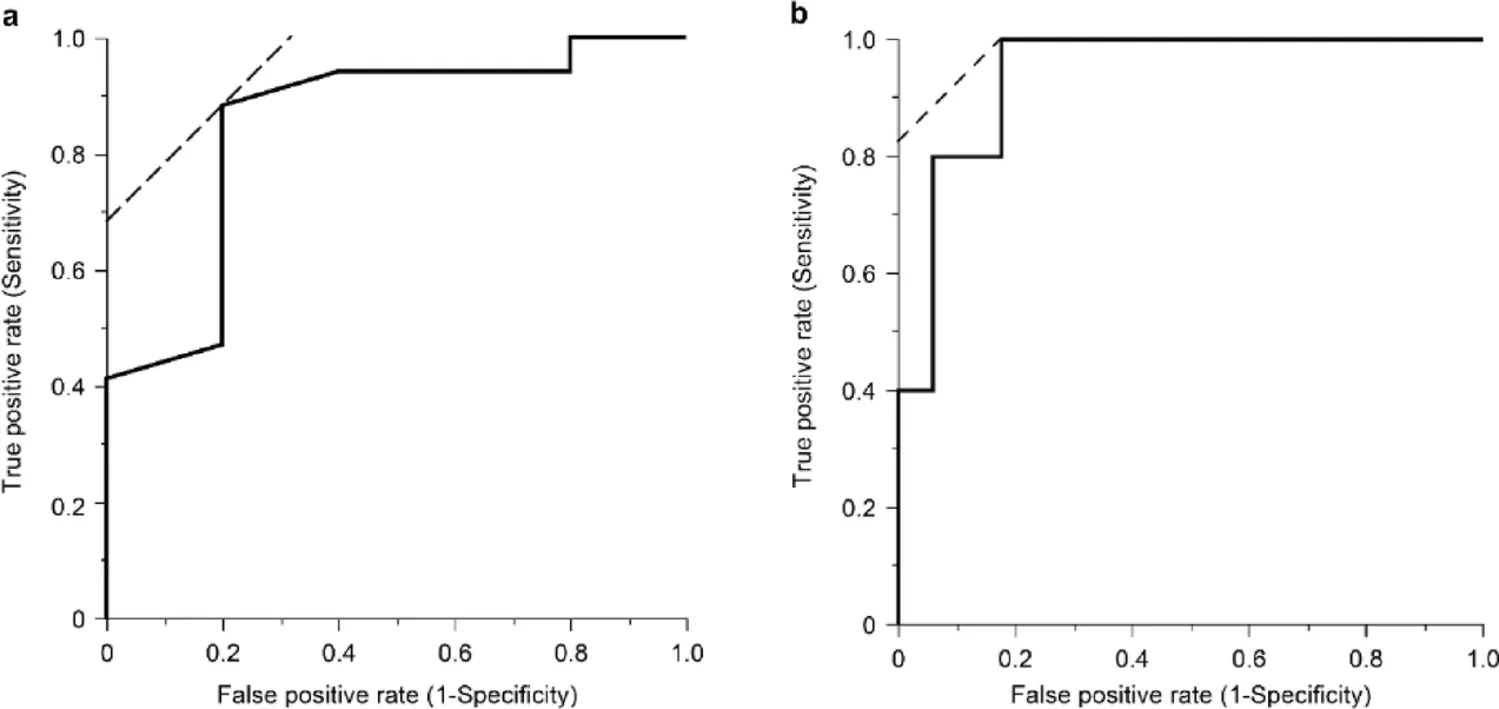

Incorrect Table 1.

**Table 1:** Clinical characteristics in fetuses diagnosed prenatally with double aortic arch

Case	Group	Sub Group	DAA type	Chromosome abnormality	Heart Complication	GA at fetal echo performed (weeks)	Age at CT performed (days)	Age at symptom onset (days)	Operative age (days)	Postoperative complication	Follow-up period (months)
1	Symptomatic	N/A	cDAA	No	No	35	3	3	5	No	32
2	Symptomatic	N/A	iDAA	No	No	35	5	4	8	No	31
3	Symptomatic	N/A	iDAA	No	No	36	5	0	7	No	26
4	Symptomatic	N/A	iDAA	No	No	35	3	5	12	No	21
5	Symptomatic	N/A	cDAA	ASXL2 mutation	Truncus, PLSVC	35	0	0	0	No	44
6	Asymptomatic	Observation	iDAA	No	No	38	4	N/A	N/A	N/A	128
7	Asymptomatic	Observation	iDAA	No	No	34	1	N/A	N/A	N/A	69
8	Asymptomatic	Observation	cDAA	No	No	35	2	N/A	N/A	N/A	36
9	Asymptomatic	Observation	cDAA	No	No	36	6	N/A	N/A	N/A	23
10	Asymptomatic	Observation	iDAA	No	No	32	30	N/A	N/A	N/A	21
11	Asymptomatic	Surgery	cDAA	22q11.2 DS	VSD	35	23	N/A	24	No	128
12	Asymptomatic	Surgery	cDAA	No	PLSVC	35	4	N/A	32	No	73
13	Asymptomatic	Surgery	iDAA	No	No	39	4	N/A	14	No	14
14	Asymptomatic	Surgery	iDAA	No	No	35	5	N/A	35	No	82
15	Asymptomatic	Surgery	cDAA	No	No	37	2	N/A	22	No	69
16	Asymptomatic	Surgery	cDAA	No	No	29	49	N/A	87	No	27
17	Asymptomatic	Surgery	cDAA	No	No	31	3	N/A	27	No	57
18	Asymptomatic	Surgery	iDAA	No	No	38	9	N/A	26	No	38
19	Asymptomatic	Surgery	cDAA	No	No	33	2	N/A	8	No	18
20	Asymptomatic	Surgery	iDAA	No	No	35	4	N/A	27	RLNP	150
21	Asymptomatic	Surgery	cDAA	No	No	34	6	N/A	25	No	12
22	Asymptomatic	Surgery	cDAA	No	No	33	8	N/A	114	No	22

*DAA* Double aortic arch, *GA* Gestational age, *CT* Not applicable, *cDAA* Complete double aortic arch, *iDAA* Incomplete double aortic arch, *PLSVC* Persistent left superior vena cava, *22q11.2 DS* 22q11.2 Deletion syndrome, *VSD* Ventricular septal defect, *RLNP* Recurrent laryngeal nerve palsy

Correct Table 1.

**Table 1:** Clinical characteristics in fetuses diagnosed prenatally with double aortic arch

Case	Group	Sub Group	DAA type	Chromosome abnormality	Heart Complication	GA at fetal echo performed (weeks)	Age at CT performed (days)	Age at symptom onset (days)	Operative age (days)	Postoperative complication	Follow-up period (months)
1	Symptomatic	N/A	cDAA	No	No	35 5/7	3	3	5	No	32
2	Symptomatic	N/A	iDAA	No	No	35 6/7	5	4	8	No	31
3	Symptomatic	N/A	iDAA	No	No	36 2/7	5	0	7	No	26
4	Symptomatic	N/A	iDAA	No	No	35 1/7	3	5	12	No	21
5	Symptomatic	N/A	cDAA	ASXL2 mutation	Truncus, PLSVC	35 6/7	0	0	0	No	44
6	Asymptomatic	Observation	iDAA	No	No	38 1/7	4	N/A	N/A	N/A	128
7	Asymptomatic	Observation	iDAA	No	No	34 3/7	1	N/A	N/A	N/A	69
8	Asymptomatic	Observation	cDAA	No	No	35 2/7	2	N/A	N/A	N/A	36
9	Asymptomatic	Observation	cDAA	No	No	36 5/7	6	N/A	N/A	N/A	23
10	Asymptomatic	Observation	iDAA	No	No	32 6/7	30	N/A	N/A	N/A	21
11	Asymptomatic	Surgery	cDAA	22q11.2 DS	VSD	35 3/7	23	N/A	24	No	128
12	Asymptomatic	Surgery	cDAA	No	PLSVC	35 1/7	4	N/A	32	No	73
13	Asymptomatic	Surgery	iDAA	No	No	39 5/7	4	N/A	14	No	14
14	Asymptomatic	Surgery	iDAA	No	No	35 1/7	5	N/A	35	No	82
15	Asymptomatic	Surgery	cDAA	No	No	37 0/7	2	N/A	22	No	69
16	Asymptomatic	Surgery	cDAA	No	No	29 5/7	49	N/A	87	No	27
17	Asymptomatic	Surgery	cDAA	No	No	31 1/7	3	N/A	27	No	57
18	Asymptomatic	Surgery	iDAA	No	No	38 5/7	9	N/A	26	No	38
19	Asymptomatic	Surgery	cDAA	No	No	33 0/7	2	N/A	8	No	18
20	Asymptomatic	Surgery	iDAA	No	No	35 4/7	4	N/A	27	RLNP	150
21	Asymptomatic	Surgery	cDAA	No	No	34 0/7	6	N/A	25	No	12
22	Asymptomatic	Surgery	cDAA	No	No	33 6/7	8	N/A	114	No	22

*DAA* double aortic arch, *GA* gestational age, *CT* computed tomography, *N/A* not applicable, *cDAA* complete double aortic arch, *iDAA* incomplete double aortic arch, *PLSVC* persistent left superior vena cava; 22q11.2 DS, 22q11.2 deletion syndrome, *VSD* ventricular septal defect, *RLNP* recurrent laryngeal nerve palsy

Incorrect Table 2.

**Table 2:** Tracheal morphometric parameters in fetuses diagnosed prenatally with double aortic arch

Case	Group	Subgroup	NTD on fetal echo (mm)	NTD Z score on fetal echo	NTA on CT (mm^2^)	RTA on CT (mm^2^)	NTA/RTA ration on CT
1	Symptomatic	N/A	1.0	-1.9	4.5	23.6	0.19
2	Symptomatic	N/A	1.1	-1.8	7.3	26.7	0.27
3	Symptomatic	N/A	1.4	-1.7	9.9	25.8	0.39
4	Symptomatic	N/A	2.1	-1.2	9.7	22.9	0.42
5	Symptomatic	N/A	0.7	-2.1	11.8	24.4	0.48
6	Asymptomatic	Observation	2.2	-1.2	18.6	25.1	0.74
7	Asymptomatic	Observation	1.9	-1.2	12.3	22.1	0.56
8	Asymptomatic	Observation	2.4	-0.9	17.9	26.6	0.67
9	Asymptomatic	Observation	2.0	-1.3	13.5	19.8	0.68
10	Asymptomatic	Observation	2.5	-0.6	23.3	28.3	0.82
11	Asymptomatic	Surgery	2.4	-0.9	28.2	30.2	0.93
12	Asymptomatic	Surgery	2.4	-0.9	22.8	29.2	0.78
13	Asymptomatic	Surgery	2.6	-1.1	19.1	32.0	0.60
14	Asymptomatic	Surgery	1.5	-1.5	11.1	23.6	0.47
15	Asymptomatic	Surgery	2.0	-1.4	14.8	21.8	0.68
16	Asymptomatic	Surgery	1.4	-1.2	11.3	25.1	0.45
17	Asymptomatic	Surgery	2.2	-0.7	17.5	23.9	0.73
18	Asymptomatic	Surgery	1.0	-2.0	13.4	21.5	0.62
19	Asymptomatic	Surgery	1.2	-1.6	8.1	23.4	0.35
20	Asymptomatic	Surgery	1.3	-1.7	11.7	22.4	0.52
21	Asymptomatic	Surgery	3.5	-0.1	16.1	31.0	0.52
22	Asymptomatic	Surgery	2.4	-0.7	27.3	33.0	0.83

*NTD* Narrowest tracheal diameter, *NTA* Narrowest tracheal area, *CT* Computed tomography, *RTA* Reference tracheal area, *N/A* Not applicable

Correct Table 2.

**Table 2:** Tracheal morphometric parameters in fetuses diagnosed prenatally with double aortic arch

Case	Group	Subgroup	NTD on fetal echo (mm)	NTD Z score on fetal echo	NTA on CT (mm^2^)	RTA on CT (mm^2^)	NTA/RTA ration on CT
1	Symptomatic	N/A	1.0	–2.2	4.5	23.6	0.19
2	Symptomatic	N/A	1.1	–2.1	7.3	26.7	0.27
3	Symptomatic	N/A	1.4	–1.9	9.9	25.8	0.39
4	Symptomatic	N/A	2.1	–1.3	9.7	22.9	0.42
5	Symptomatic	N/A	0.7	–2.4	11.8	24.4	0.48
6	Asymptomatic	Observation	2.2	–1.4	18.6	25.1	0.74
7	Asymptomatic	Observation	1.9	–1.4	12.3	22.1	0.56
8	Asymptomatic	Observation	2.4	–1.0	17.9	26.6	0.67
9	Asymptomatic	Observation	2.0	–1.5	13.5	19.8	0.68
10	Asymptomatic	Observation	2.5	–0.7	23.3	28.3	0.82
11	Asymptomatic	Surgery	2.4	–1.1	28.2	30.2	0.93
12	Asymptomatic	Surgery	2.4	–1.0	22.8	29.2	0.78
13	Asymptomatic	Surgery	2.6	–1.3	19.1	32.0	0.60
14	Asymptomatic	Surgery	1.5	–1.8	11.1	23.6	0.47
15	Asymptomatic	Surgery	2.0	–1.5	14.8	21.8	0.68
16	Asymptomatic	Surgery	1.4	–1.4	11.3	25.1	0.45
17	Asymptomatic	Surgery	2.2	–0.8	17.5	23.9	0.73
18	Asymptomatic	Surgery	1.0	–2.3	13.4	21.5	0.62
19	Asymptomatic	Surgery	1.2	–1.8	8.1	23.4	0.35
20	Asymptomatic	Surgery	1.3	–1.9	11.7	22.4	0.52
21	Asymptomatic	Surgery	3.5	–0.1	16.1	31.0	0.52
22	Asymptomatic	Surgery	2.4	–0.9	27.3	33.0	0.83

*NTD* narrowest tracheal diameter, *NTA* narrowest tracheal area, *CT* computed tomography, *RTA* reference tracheal area, *N/A* not applicable

The authors would like to apologize for any inconvenience caused.

## Data Availability

No datasets were generated or analysed during the current study.

